# Gamma-glutamyl transferase: risk and prognosis of cancer

**DOI:** 10.1038/bjc.2012.128

**Published:** 2012-04-24

**Authors:** I S Fentiman

**Affiliations:** 1 GKT School of Medicine, Guy’s Hospital, London SE1 9RT, UK

Gamma-glutamyl transferase (GGT) is a membrane-bound enzyme catabolising reduced glutathione to cysteine and glycine in Meister’s *γ*-glutamyl cycle (Orlowski and Meister, 1970). This delivers cysteine for intracellular synthesis of glutathione, the major thiol anti-oxidant. Elevated serum levels of GGT are markers of oxidative stress, resulting from factors including alcohol, heavy metals, cardiovascular disease and diabetes.

In this issue, an Austrian multicentre study is reported, which shows an association between GGT and prognosis in women with endometrial carcinoma (Seebacher *et al*, 2012). Gamma-glutamyl transferase levels were evaluated in 874 consecutive patients who were stratified in risk groups. After multivariate analysis, elevated and highly elevated serum GGT levels were independently associated with poorer survival. There was no association with advanced tumour stage, higher tumour grade or with more aggressive histology.

High levels of GGT seem to increase the risk of progression of high-grade cervical dysplasia to invasive carcinoma. Recently, as part of a multi-centre trial, pre-treatment GGT levels were examined in 692 patients with cervical cancer (Polterauer *et al*, 2011). Gamma-glutamyl transferase serum levels were significantly associated with FIGO stage and age (but not with lymph node involvement (*P*=0.85), and histological type. There was a linear correlation between GGT and prognosis.

Furthermore, higher serum levels of GGT, within the normal range, are associated with an increased cancer risk. In the Vorarlberg study of 92 983 females, there was an increasing hazard rate for various cancers with higher levels of the enzyme (Strasak *et al*, 2008). Within the Guernsey Cohort Study, a highly significant association was found between levels of GGT and breast cancer risk in premenopausal women (Fentiman and Allen, 2010). These results were confirmed in the Apolipoprotein Mortality Risk (AMORIS) study of 545 460 Swedes (Van Hemelrijck *et al*, 2011). When subdivided by categories of GGT (<18, 18–36, 36–72, >72 U l^–1^) there was an increasing association with overall cancer risk. For those with glucose levels >6.11 mmol l^–1^, the association with risk of prostate, breast and liver cancer became stronger, supporting a role for oxidative stress in the process of carcinogenesis.

In the third US National Health and Nutrition Examination Survey (NHANES) blood lead and urinary cadmium levels were measured together with serum GGT in 10 098 adults (Lee *et al*, 2006). After adjustment for race, sex and age, both blood lead and urinary cadmium levels showed graded positive associations, with serum GGT. Subsequently, elevated urinary cadmium levels were shown to be associated with increased breast cancer risk (Gallagher *et al*, 2010). This strong association with a marker of oxidative stress in a normal population suggests a possible carcinogenic role for lead and cadmium in individuals with low environmental exposure.

Death certificate-based 12-year mortality was also examined in the third NHANES project in relation to serum alanine amino-transaminase (ALT) and GGT (Ruhl and Everhart, 2009). Elevated ALT was associated with deaths from hepatic disease but not with all-cause mortality. In contrast, elevated GGT was associated with all-cause mortality from liver disease, neoplasms and diabetes.

There is evidence that GGT is dysregulated in malignant cells and by producing reactive oxygen species cause tumour progression towards more aggressive phenotypes associated with a worse prognosis (Dawson *et al*, 1979, Hanigan *et al*, 1999). In a study of human GGT-transfected melanoma cells, higher levels of GGT activity were associated with greater levels of background DNA damage and oxidised bases (Corti *et al*, 2008). This was unrelated to differences in cell cycle distribution or apoptotic rates. Culture of GGT-over-expressing cells with GGT substrates and catalytic iron resulted in further DNA damage, eliminated in the presence of GGT inhibitors such as L-2-amino-4-boronobutanoic acid (ABBA).

Curcumin, a polyphenol extracted from *Curcuma longa* has anti-tumour effects on ZR-75-1 cells, resistant to oxidative damage (Quiroga *et al*, 2010). In a dose-dependent manner, it is both cytotoxic and inhibitory to GGT activity. If elevated levels of GGT are marking an increased risk of recurrence of cancer, it is possible that curcumin has therapeutic potential in this situation. Under other circumstances the agent has been shown to be both effective and non-toxic in a randomised double-blind placebo-controlled trial of Iranian war veterans with sulphur mustard-induced pruritus (Panahi *et al*, 2011).

So, what can be made of this? In terms of carcinogenesis, GGT clearly is a marker of oxidative stress. What is unclear is whether GGT has a direct aetiological role or is an indicator of collateral damage. Another factor to be considered is that GGT is elevated by alcohol consumption, which has a direct role in carcinogenesis for liver, pancreas, larynx and breast. It should, however, be noted that higher levels of GGT within the normal range are associated with increased risk of cancer so that causes other than alcohol may be involved. More basic studies are needed using mice with GGT knockout to determine whether GGT has an aetiological role in carcinogenesis or is a mere marker of damage. In patients who have been treated for cancer, higher levels of GGT may signal ongoing oxidative stress. The role of non-specific inhibitors of GGT such as curcumin is potentially interesting but what we need is a specific non-toxic GGT inhibitor that can be tested in a wider preventive/adjuvant role in selected patients with premalignant changes or established cancer.
